# Supplementary material for: Comparative Analysis of Acanthopanacis Cortex and Periplocae Cortex Using an Electronic Nose and Gas Chromatography–Mass Spectrometry Coupled with Multivariate Statistical Analysis
Source: Molecules. 2022 Dec 16;27(24):8964. doi: 10.3390/molecules27248964 (PMC9781861; doi:10.3390/molecules27248964)
Supplement: Supplementary file 1 [file molecules-27-08964-s001.zip › molecules-2088197-supplementary.pdf]

Supplementary Materials:

Table S1. Details of samples of Acanthopanacis Cortex and Periplocae Cortex.

No.	Sample Name	Batch Code	Origin	Collection place
W-01	Acanthopanacis Cortex	20201001	Jilin Province	Drug store, Jingmen, Hubei
W-02	Acanthopanacis Cortex	201907001	Hebei Province	Drug store, Xiangyang, Hubei
W-03	Acanthopanacis Cortex	180101	Hubei Province	Drug store, Xiangyang, Hubei
W-04	Acanthopanacis Cortex	2020903100200	Hubei Province	Drug store, Xiangyang, Hubei
W-05	Acanthopanacis Cortex	Not marked	Not marked	Drug store, Xiangyang, Hubei
W-06	Acanthopanacis Cortex	202101001	Hunan Province	Drug store, Xiangyang, Hubei
W-07	Acanthopanacis Cortex	Not marked	Hubei Province	Drug store, Xiangyang, Hubei
W-08	Acanthopanacis Cortex	2110210011	Hubei Province	Hospital, Xiangyang, Hubei
W-09	Acanthopanacis Cortex	20200601	Hubei Province	CHMs company, xiangyang, Hubei
W-10	Acanthopanacis Cortex	Not marked	Not marked	Drug store, Xiangyang, Hubei
W-11	Acanthopanacis Cortex	210801	Shanxi Province	Medicine Market, Bozhou, Anhui
W-12	Acanthopanacis Cortex	210101	Shannxi Province	Medicine Market, Bozhou, Anhui
W-13	Acanthopanacis Cortex	200801	Shanxi Province	Medicine Market, Bozhou, Anhui
W-14	Acanthopanacis Cortex	201102	Shannxi Province	Medicine Market, Bozhou, Anhui
W-15	Acanthopanacis Cortex	201101	Shannxi Province	Medicine Market, Bozhou, Anhui
W-16	Acanthopanacis Cortex	160405	Anhui Province	Drug store, Xiangyang, Hubei
X-01	Periplocae Cortex	202004001	Hebei Province	Drug store, Xiangyang, Hubei
X-02	Periplocae Cortex	Not marked	Henan Province	Drug store, Xiangyang, Hubei
X-03	Periplocae Cortex	Not marked	Not marked	Hospital, Xiangyang, Hubei
X-04	Periplocae Cortex	Not marked	Not marked	Drug store, Xiangyang, Hubei
X-05	Periplocae Cortex	210501	Shannxi Province	Medicine Market, Bozhou, Anhui
X-06	Periplocae Cortex	210401	Shannxi Province	Medicine Market, Bozhou, Anhui
X-07	Periplocae Cortex	200901	Shanxi Province	Medicine Market, Bozhou, Anhui
X-08	Periplocae Cortex	200801	Shannxi Province	Medicine Market, Bozhou, Anhui
X-09	Periplocae Cortex	201101	Not marked	Medicine Market, Bozhou, Anhui
X-10	Periplocae Cortex[a]	20160501	Hubei Province	CHM company, xiangyang, Hubei[a]
X-11	Periplocae Cortex[b]	170625	Anhui Province	Clinic, Xiangyang, Hubei[b]
X-12	Periplocae Cortex[b]	171203	Anhui Province	Drug store, Xiangyang, Hubei[b]
X-13	Periplocae Cortex[c]	Not marked	Hubei Province	Drug store, Xiangyang, Hubei[c]

[a] Retention samples of the supervision and inspection samples in 2019 (Sample name marked Acanthopanacis Cortex);
[b] Retention samples of the supervision and inspection samples in 2018 (Sample name marked Acanthopanacis Cortex);
[c] Samples for supervision and inspection in 2021 (Sample name marked Acanthopanacis Cortex).

Table S2. Sensors used and their main applications in PEN3 E-nose.

Sensor number	Sensor name	General description
1	W_1C	Aromatic compounds
2	W_5S	Very sensitive, broad range of sensitivity, reacts to nitrogen oxides, very sensitive with negative signals
3	W_3C	Ammonia, used as sensor for aromatic compounds
4	W_6S	Mainly hydrogen
5	W_5C	Alkanes, aromatic compounds, less polar compounds
6	W_1S	Sensitive to methane. Broad range
7	W_1W	Reacts on sulfur compounds, H_2S. Otherwise sensitive to many terpenes and sulphur organic compounds
8	W_2S	Detects alcohol's, partially aromatic compounds, broad range
9	W_2W	Aromatic compounds, sulphur organic compounds
10	W_3S	Reacts on high concentrations (> 100 ppm) of methane-aliphatic compounds

Table S3. *P* value of volatile organic compounds (VOCs) with VIP > 1 in OPLS-DA model analysis.

No.	VOCs	Formula	VIP	P
33	2-Hydroxy-4-methoxybenzaldehyde	$C_8H_8O_3$	1.62062	< 0.05
16	(1R,5S)-6,6-dimethyl-bicyclo[3.3.1]hept-2-en-2-carbaldehyde	$C_{10}H_{14}O$	1.49172	< 0.05
68	methyl 2-hydroxy-4-methoxybenzoate	$C_9H_{10}O_4$	1.48045	< 0.05
8	(1S,5S)-7,7-dimethyl-4-methylidenebicyclo[3.1.1]heptan-3-ol	$C_{10}H_{16}O$	1.44243	< 0.05
20	(1R,5S)-2-methyl-5-prop-1-en-2-ylcyclohex-2-en-1-ol	$C_{10}H_{16}O$	1.43818	< 0.05
25	2,7,7-trimethylbicyclo[3.1.1]hept-2-en-4-one	$C_{10}H_{14}O$	1.43137	< 0.05
12	2-(4-methyl-1-cyclohexa-2,4-dienyl)propan-2-ol	$C_{12}H_{20}O_3$	1.3873	< 0.05
7	1-(2,2,3-trimethyl-1-cyclopent-3-enyl)ethanone	$C_{10}H_{16}O$	1.37597	< 0.05
15	2-[(1R)-4-methyl-1-cyclohex-3-enyl]propan-2-ol	$C_{10}H_{18}O$	1.35918	< 0.05
19	(1S,5S)-2,7,7-trimethylbicyclo[3.1.1]hept-2-en-4-one	$C_{10}H_{14}O$	1.34432	< 0.05
10	2-(4-methylidene-1-cyclohex-2-enyl)propan-2-ol	$C_{12}H_{20}O_3$	1.3365	< 0.05
66	5-formyl-2-methoxyphenyl acetate	$C_{10}H_{10}O_4$	1.29551	< 0.05
24	(5S)-2-methyl-5-prop-1-en-2-ylcyclohex-2-en-1-one	$C_{10}H_{14}O$	1.27463	< 0.05
62	3,4,5-trimethyloxolan-2-one	$C_7H_{12}O_2$	1.27007	< 0.05
11	7,7-dimethyl-4-methylidenebicyclo[3.1.1]heptan-3-one	$C_{10}H_{14}O$	1.25103	< 0.05
13	(1R)-4-methyl-1-propan-2-ylcyclohex-3-en-1-ol	$C_{10}H_{18}O$	1.23982	< 0.05
14	2-(4-methylphenyl)propan-2-ol	$C_{10}H_{14}O$	1.23523	< 0.05
17	Dodecane	$C_{12}H_{26}$	1.21744	< 0.05
51	(1aR,4aR,7S,7aR,7bR)-1,1,7-Trimethyl-4-methylenedecahydro -1H-cyclopropa[e]azulen-7-ol	$C_{15}H_{24}O$	1.21026	< 0.05
58	5-(5,5,8a-trimethyl-2-methylidene-3,4,4a,6,7,8-hexahydro-1H-naphthalen-1-yl)-3-methylpent-1-en-3-ol	$C_{20}H_{34}O$	1.19979	< 0.05

71	2,4-ditert-butylphenol	$C_{14}H_{22}O$	1.16325	< 0.05
52	(1R,4R,6R,10S)-4,12,12-Trimethyl-9-methylene-5-oxatricyclo[8.2.0.0]dodecane	$C_{15}H_{24}O$	1.14638	< 0.05
29	[(4S)-4-prop-1-en-2-yl-1-cyclohexenyl]methanol	$C_{10}H_{16}O$	1.13892	< 0.05
64	3-Hydroxy-4-methoxybenzaldehyde	$C_8H_8O_3$	1.05977	< 0.05
